# Using serological measures to monitor changes in malaria transmission in Vanuatu

**DOI:** 10.1186/1475-2875-9-169

**Published:** 2010-06-16

**Authors:** Jackie Cook, Heidi Reid, Jennifer Iavro, Melissa Kuwahata, George Taleo, Archie Clements, James McCarthy, Andrew Vallely, Chris Drakeley

**Affiliations:** 1 London School of Hygiene & Tropical Medicine, Keppel Street, London WC1E 7HT, UK; 2 School of Population Health, University of Queensland, Herston, Qld 4006, Australia; 3 Ministry of Health, Port Vila, Vanuatu; 4 Queensland Institute of Medical Research, Herston, Qld 4006, Australia; 5 School of Medicine, University of Queensland, Herston, Qld 4006, Australia

## Abstract

**Background:**

With renewed interest in malaria elimination, island environments present unique opportunities to achieve this goal. However, as transmission decreases, monitoring and evaluation programmes need increasingly sensitive tools to assess *Plasmodium falciparum *and *Plasmodium vivax *exposure. In 2009, to assess the role of serological markers in evaluating malaria transmission, a cross-sectional seroprevalence study was carried out in Tanna and Aneityum, two of the southernmost islands of the Vanuatu archipelago, areas where malaria transmission has been variably reduced over the past few decades.

**Methods:**

Malaria transmission was assessed using serological markers for exposure to *P. falciparum *and *P. vivax*. Filter blood spot papers were collected from 1,249 people from Tanna, and 517 people from Aneityum to assess the prevalence of antibodies to two *P. falciparum *antigens (MSP-1_19 _and AMA-1) and two *P. vivax *antigens (MSP-1_19 _and AMA-1). Age-specific prevalence was modelled using a simple catalytic conversion model based on maximum likelihood to generate a community seroconversion rate (SCR).

**Results:**

Overall seropositivity in Tanna was 9.4%, 12.4% and 16.6% to *P*. *falciparum *MSP-1_19_, AMA-1 and Schizont Extract respectively and 12.6% and 15.0% to *P. vivax *MSP-1_19 _and AMA-1 respectively. Serological results distinguished between areas of differential dominance of either *P. vivax *or *P. falciparum *and analysis of age-stratified results showed a step in seroprevalence occurring approximately 30 years ago on both islands, indicative of a change in transmission intensity at this time. Results from Aneityum suggest that several children may have been exposed to malaria since the 2002 *P. vivax *epidemic.

**Conclusion:**

Seroepidemiology can provide key information on malaria transmission for control programmes, when parasite rates are low. As Vanuatu moves closer to malaria elimination, monitoring changes in transmission intensity and identification of residual malaria foci is paramount in order to concentrate intervention efforts.

## Background

Malaria elimination is once again a stated goal for a number of malaria-endemic countries [1]. In areas where transmission intensity is low, malaria control programmes are considering switching from sustained control to elimination. Monitoring of malaria transmission intensity (MTI), and targeting interventions to settlements experiencing higher malaria transmission will be vital in this endeavour [2]. Interventions in geographically isolated areas, such as islands, may be more successful due to better control of imported transmission, geographically defined areas to target, and high community participation [3,4]. Sustained control efforts and monitoring in these areas can lead to the successful eradication of malaria, such as occurred in Mauritius in the 1960s [5].

However, areas of low transmission, or areas where transmission has been reduced substantially, pose considerable challenges for monitoring and evaluation. Exposure to infection can be markedly heterogeneous which can be an important determinant of the rate of progress, and thus the time required to achieve elimination [6]. When transmission is low, traditional measures of MTI such as the entomological inoculation rate (EIR) and parasite rates (PR) lack sensitivity because numbers of positive samples (mosquito and human) are low. Additionally, the low frequency of positive samples represents a formidable logistical challenge, and further, both entomological and parasitological measures are affected by seasonality [7-9]. An alternative measure for MTI is to calculate the prevalence of anti-malaria antibodies in the local population. Serological markers of transmission show greater sensitivity in low transmission areas, as seroprevalence reflects cumulative exposure and thus is less affected by seasonality due to the longer duration of specific antibody responses. Sample collection and analysis can be readily scaled up, and analysis is relatively simple, making this approach adaptable for resource poor settings [10].

Seroepidemiological studies have previously been used to assess malaria transmission intensity [11], reductions in transmission [12-14] and malaria eradication [5,15,16]. However, variation in source of antigen and the subjectivity of the detection methods led to this method falling out of favour [17]. The availability of specific recombinant malarial antigens, and the development of standardized, sensitive enzyme-linked immunosorbent assays (ELISA) mean that seroepidemiological study has once again become an attractive methodology, both for assessment of malaria transmission and for assessing changes in prevalence following the implementation of control programmes [10,18-21].

**Figure 1:**
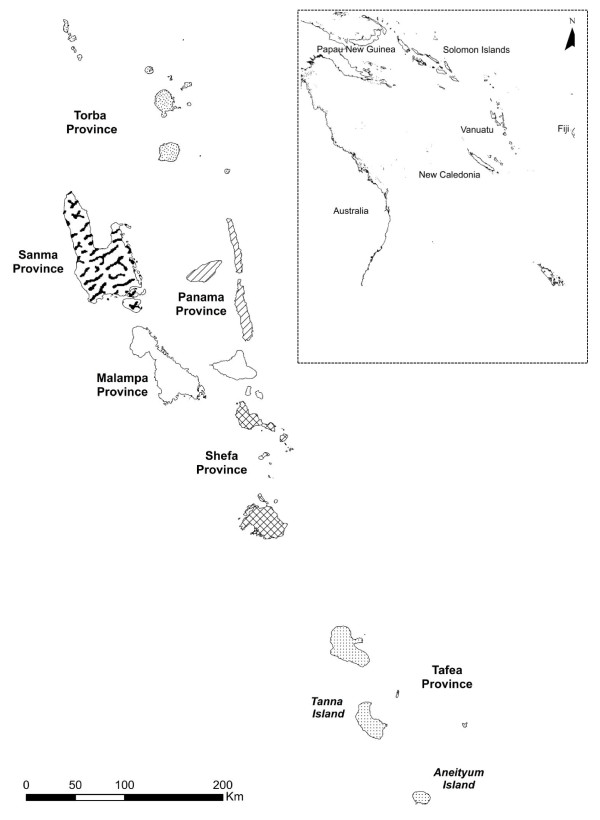
**Location of Tanna and Aneityum, Vanuatu in the South West Pacific**. Map of Vanuatu showing the location of Tafea Province within the country and the location of Vanuatu with respect to neighbouring countries in the Western Pacific region (inset).

The utility of serological markers as a methodology for describing malaria exposure was investigated as part of the evaluation of malaria elimination efforts in Vanuatu. Vanuatu is a South Pacific archipelago made up of over 80 islands, each with varying levels of malaria transmission. There have been several large-scale control programmes implemented in the country over the last 50 years and a reduction in reported cases has been recorded over the last decade (Ministry of Health, Vanuatu). In March 2008, the Vanuatu government revised the goal of their National Malaria Programme (NMP) from control to elimination, with the aim to eliminate malaria from Tafea, the most southerly province, by 2015 (Figure 1). The most southerly island of the province, Aneityum (Population *c*.800), has been subject to extensive malaria control programmes. There were no reported malaria cases on Aneityum between 1991 and 2002, when a *P*. *vivax *epidemic occurred [3]. Since 2002, no more cases have been reported. Tanna, the second largest of the islands in the province, (Population *c*.20,000) is characterized by low but continuing *P. falciparum *and *P. vivax *transmission. A malariometric study performed in 2008 revealed low level parasitaemia and mainly coastal transmission for both species [22].

This paper reports on the sero-epidemiological results from samples collected from Tanna and Aneityum in May 2009, with the aim of assessing the utility of specific serological tools for both ongoing monitoring of MTI, and assessment of changes in MTI, in the pre- and post-elimination era.

## Methods

### Study site and population

Tanna and Aneityum are in Tafea Province, in southern Vanuatu (Figure 1). The main occupation in the province tends to be subsistence farming which takes place year round. Group meetings take place in *Nakamals *(central meeting place), and men often convene (often in the evenings) to drink kava (a sedating drink) in these areas. Knowledge of malaria is predominantly high, although community adherence to protective measures can be low where the threat is perceived as minimal[23]. Rainfall is seasonal, with a wet season from January to May. The only known malaria vector is *Anopheles farauti *[3]. In 1988, country-wide distribution of insecticide-treated nets (ITNs) began, following the cessation of indoor residual spraying (IRS)[24].

#### Tanna

**Figure 2:**
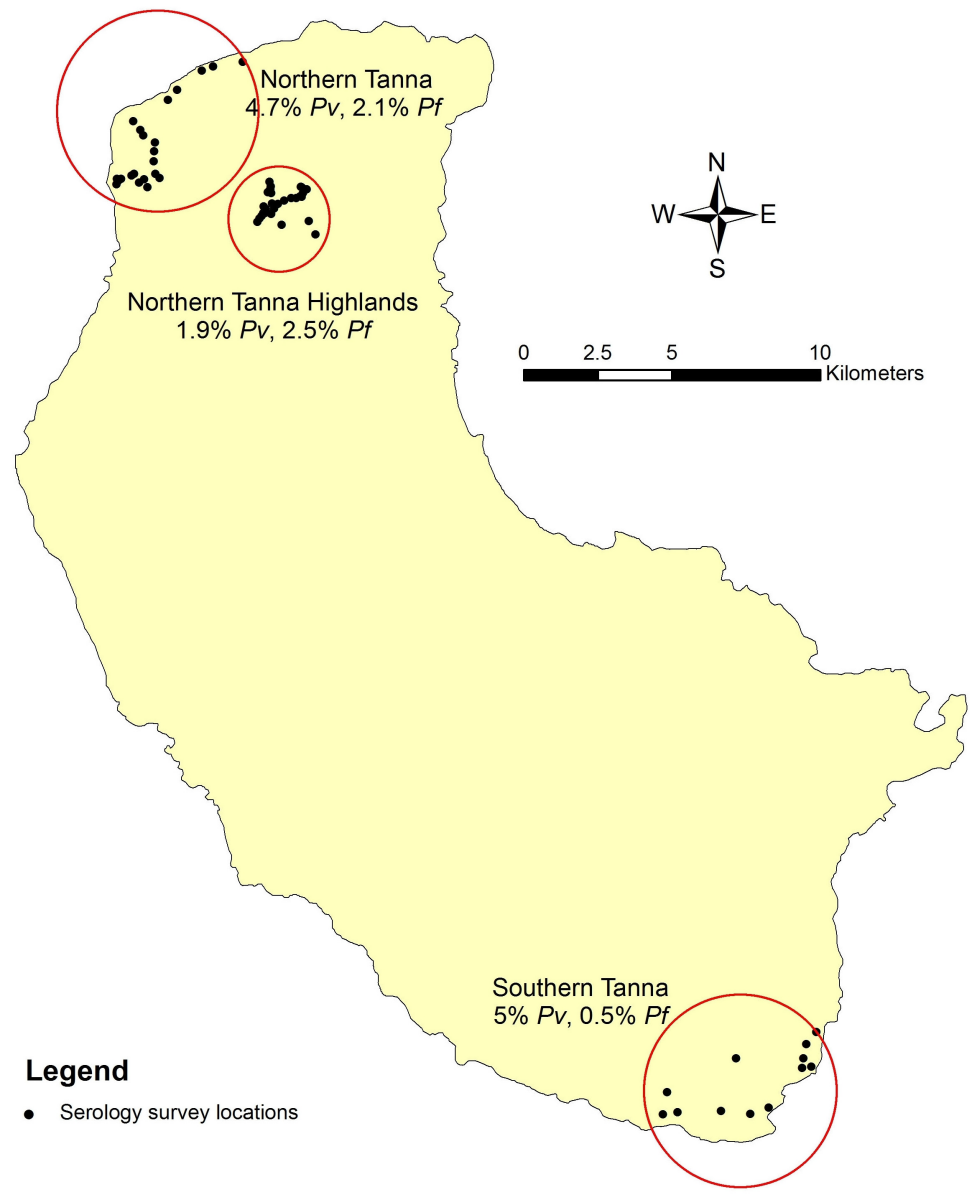
**Summary of Plasmodium speciation PCR results obtained from Tanna in 2008**. PCR results for children over 2 and under 15 are presented, adapted from data presented in Reid et al [22]. Each black spot represents the home settlement of at least one child involved in the survey. Children were sampled from a total of 44 villages across three settlements on Tanna.

Tanna has a population of approximately 20,000 (1999 census). Both *P. falciparum *and *P. vivax *malaria occur on Tanna. In 2008, a prevalence study using species specific PCR of samples obtained from school children identified three main areas of malaria transmission [22] (Figure 2). Briefly, Northern Tanna (Hebron/Lounapil) had the highest malaria prevalence with 2.1% (10/486) and 4.7% (23/486) positive for *P. falciparum *and *P*. *vivax *respectively; the Northern highlands had the lowest with 2.5% (12/481) and 1.9% (9/481) for *P. falciparum *and *P. vivax *respectively; and the Southern Coast of Tanna (Etakua/Kwamera) had very low levels of *P. falciparum *(0.5%, 3/576) but higher levels of *P. vivax *(5.0%, 29/576). These figures are summarized in Figure 2. Communities living in these settlements were asked to participate in the seroepidemiological study.

#### Aneityum

Aneityum lies to the south of Tanna and is much smaller. The island is sparsely inhabited with a population of approximately 800 distributed in three main settlements. Due to weather constraints during the survey, samples were only collected from two of the three settlements; Anel and Port Patrick. Results for these two settlements have been combined for the majority of the analyses. In Aneityum, weekly mass drug administration (using Primaquine with chloroquine and pyrimethamine with sulphadoxine) was carried out for 9 weeks in 1991. Larvivorous fish were simultaneously introduced into identified breeding sites of *An. farauti *[3]. No parasites have been detected by microscopy in annual surveys since 2003 (A. Kaneko; personal communication).

### Survey methodology

The cross-sectional survey took place in May 2009. Participants were recruited to the study by the Ni-Vanuatu malaria control team. Village leaders were sensitized to the study by trained field workers and together provided information to community members at community meetings. Community members willing to participate were asked to complete a written Informed Consent Form by providing a signature or witnessed thumbprint. A central meeting point was chosen (*nakamal*, or a school or community centre) and families were invited from surrounding villages to be part of the survey. A cross-section of the population was sampled, with high levels of community participation. Demographic data were collected for each person, including age, sex and details on whether they owned a bed net, and had used a bed net the previous night. Participants were tested with a rapid diagnostic test (ICT COMBO cassette) if they presented with a fever and were treated accordingly if the test was positive. Finger prick blood samples were collected on labelled Whatman 3 mm filter paper, allowed to air dry and then packaged in sealable bags containing silica gel, before being transported for analysis in Brisbane, Australia.

### Assay of anti-malarial antibodies

Antibodies were eluted from filter paper blood spots (FPBS) and assayed by ELISA as previously described [19]. Briefly, a 3.5 mm circle was cut from the spot and placed into 300 μl of phosphate buffered saline with 0.5% tween20 (PBS-T) and 0.05% sodium azide, approximately equivalent to a 1/200 serum dilution. All samples were tested for the presence of human antibodies (IgG) against merozoite surface protein-1_19 _(MSP-1_19_) and apical membrane antigen-1 (AMA-1) of *P. falciparum *(MSP-1_19_- Wellcome strain, AMA-1-3D7) and *P. vivax *(MSP-1_19_-Belem strain, AMA-1 (Sal-1). Samples were also tested on freeze thawed *P. falciparum *Schizont Extract (concentration of 1 × 10^8^/ml) which was coated onto plates at 1/500. Briefly, each antigen was coated on high absorbance plates (Immunlon4) at 0.5 μg/ml and stored at 4°C overnight. The plate was washed three times in PBS-T and blocked for 3 hours with 1% (w/v) milk powder. Following three more washes, samples were added in duplicate at a final dilution of 1/1000. A pool of sera from a highly endemic area in Africa was titrated on each plate as a positive control. The following day, following five washes, anti-human IgG HRP (DAKO) was incubated in each plate for three hours. The plates were then developed using OPD (Sigmafast) for 20 minutes in the dark. Plates were read at 492 nm on a Molecular Devices Versa Max ELISA reader.

### Statistical methods

The serology survey data was double entered into separate Microsoft Excel spreadsheets. The completed spreadsheets were converted to Microsoft Access databases which were compared using the FLG comparison script [25] which yielded 1.67% differences in entry that were corrected and checked again. The resulting double entered database was logically validated for value homogeneity and range checking, using scripts developed in SAS/STAT software (v1.93 for Windows). The cleaned database was then converted back to Microsoft Excel for analysis in STATA (v.10).

Samples where duplicate optical densities (ODs) had more than 20% variation between them were excluded from the analysis. Raw ODs were transformed into titres using the standard curve to normalize between plates and dichotomized into positive or negative using a mixture model, as described previously [19]. Similar models have been used for defining cut offs for the Mantoux test for tuberculosis where pre-exposure to environmental bacteria can cause non-specific reactions [26] and also in serological tests for measles and rubella [27,28]. Briefly, the data were fitted using maximum likelihood methods to establish seronegative and seropositive Gaussian distributions. For each antigen, the cut off was then set as the mean titre of the seronegative distribution plus three standard deviations [20].

A simple reversible catalytic model was fitted to the dichotomized data using maximum likelihood methods [18]. The model generates a seroconversion rate (SCR or λ) and a seroreversion rate (ρ). For the purposes of this study the seroreversion rate was not fixed and allowed to vary as determined by the model. The model was used to generate age seroprevalence curves, from which a seroconversion rate (SCR) representing the force of infection for the community was calculated. If visual examination of SCR suggested it was not uniform over the whole population (i.e. there was an obvious step in the age seroprevalence data), models allowing for two forces of infection profile and profile likelihood plots were run to determine when the most likely time for change in transmission intensity occurred (or at what age the step in seroprevalence occurs) [20]. These resulted in a predicted time of change in transmission (and 95% confidence intervals), which was subsequently incorporated into the catalytic model to generate estimates for previous and current SCR. Models allowing for two forces of infection were preferred if the fit compared to the single force model was significantly better by likelihood ratio (LR) test at a p < 0.05.

The PfSE antigen is multi-antigenic and not necessarily specific for *P. falciparum *antigens as many of the same proteins are likely to be present in other species of *Plasmodium *and possibly in other infections. Therefore, the analysis primarily focuses on the results for the specific antigens analysed. Village of residence was mapped based on coordinates recorded the previous year [22]. Mean seroprevalence in children (to represent recent and local transmission) aged between 1 and 10 (inclusive) was plotted by village using ArcGIS (v.9.3.1). Logistic regression was used to compare differences in seroprevalence between settlements, whilst controlling for age group.

### Ethical approval

This research was approved by the Vanuatu Ethics Committee, Ministry of Health, Vanuatu and by the University of Queensland.

## Results

A total of 364, 515 and 370 people were recruited from North Tanna, the Northern Tanna Highlands and Southern Tanna respectively. Five hundred and seventeen people were recruited from Aneityum from two of the three villages. Fewer men were sampled than women (854/906) but this difference was not significant, and the ratio did not vary across clusters (Chi^2 ^p = 0.87). Sampling often took place in schools, meaning that children of school age were more heavily sampled- the age distribution varied slightly depending on the cluster (Table 1). In total, 65% of people sampled were under the age of 20, a figure that is higher than the 55% national distribution [29].

**Table 1:** Population demographics of survey participants (%(n))

		Northern Tanna (n = 364)	Northern Tanna Highlands (n = 515)	Southern Tanna (n = 370)	Aneityum (n = 517)	Total (n = 1766)
	**Male**	47.3 (172)	48.1 (245)	50.3 (186)	48.6 (251)	48.5 (854)
**Sex**	**Female**	52.8 (192)	51.9 (264)	49.7 (184)	51.5 (266)	51.5 (906)
	**Not recorded**	0	6	0	0	6

	**0-5**	18.4 (67)	23.1 (119)	14.1 (52)	17.4 (90)	18.6 (328)
	**5-20**	48.4 (176)	42.9 (221)	52.7 (195)	42.4 (219)	45.9 (811)
**Age (Years)**	**20-80**	33.2 (121)	34.0 (175)	33.2 (123)	40.2 (208)	35.5 (627)
	**Total**	100 (364)	100 (515)	100 (370)	100 (517)	100 (1766)

	**No**	21.8 (79)	61.7 (269)	11.1 (41)	10.1 (52)	26.1 (441)
**Used a bed net the night before the survey**	**Yes**	78.2 (284)	38.3 (167)	88.9 (329)	89.9 (465)	73.8(1245)
	**Not recorded**	1	79	0	0	80

Due to current malaria control activities, ownership and reported use of bed nets was generally high, with usage close to 80% in Northern Tanna, Southern Tanna and Aneityum (Table 1). However in the Northern highlands in Tanna the reported usage of nets was lower, with only 38% (167/436) of survey respondents reporting to have used the nets the night before despite 98% (503/512) reporting they owned nets (Table 1). This is likely to be a result of the perceived low malaria risk in this area[23].

### Antibody responses in the population

**Figure 3:**
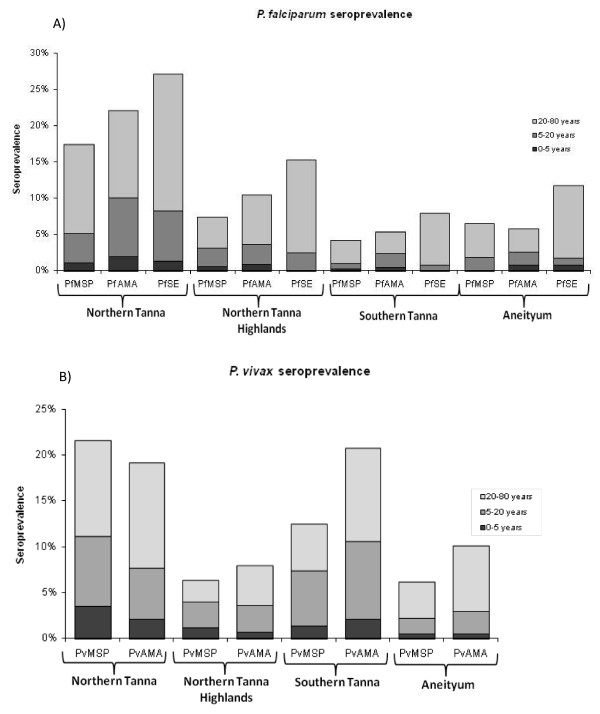
Seroprevalence to *P.falciparum *and *P.vivax *antigens in 3 settlements from Tanna and from two settlements combined on Aneityum, by three age groups

Seroprevalence for each settlement, by age group, is summarized in Table 2 and Figure 3. Overall, seroprevalence increased with age and was higher to AMA-1 antigens than to MSP-1_19 _antigens. Seroprevalence was highest to PfSE. There was no difference in seroprevalence between males and females for any of the antigens tested (p > 0.1 for all antigens). Overall, seropositivity in Tanna was 9.4% and 12.4% and 16.6% for *P. falciparum *MSP-1_19_, AMA-1 and PfSE respectively and 12.6% and 15.0% for *P. vivax *MSP-1_19 _and AMA-1 respectively. In Aneityum, seropositivity was 6.6%, 5.8% and 11.8% for *P. falciparum *MSP-1_19_, AMA-1 and PfSE respectively and 6.2% and 10.1% for *P. vivax *MSP-1_19 _and AMA-1 respectively.

**Table 2:** Seroprevalence to four malarial antigens by age group

Location	Age group (years)	N Pf MSP	Pf MSP +ve	% Pf MSP +ve	N Pf AMA	Pf AMA +ve	% Pf AMA +ve	N Pf SE	Pf SE +ve	% PfSE +ve	N Pv MSP	Pv MSP +ve	% Pv MSP +ve	N Pv AMA	Pv AMA +ve	% Pv AMA+ve
	0-5	64	4	**6.3 (1.6-12.3)**	65	7	**10.8 (3.0-18.5)**	66	5	**7.6 (1.0-14.1)**	66	13	**19.7 (9.8-29.5)**	65	8	**12.3 (4.1-20.5)**
**Northern Tanna**	5-20	176	15	**8.5 (4.4-12.7)**	174	29	**16.7 (11.1-22.3)**	176	25	**14.2 (9.0-19.4)**	172	27	**15.7 (10.2-21.2)**	175	20	**11.4 (6.7-16.2)**
	20-80	121	44	**36.4 (27.7-45.1)**	119	43	**36.1 (27.4-45.0)**	120	68	**56.7 (47.7-65.7)**	119	37	**31.1 (22.7-39.5)**	121	41	**33.9 (25.3-42.4)**

	Total	361	63	**17.5 (13.5-21.4)**	358	79	**22.1 (17.8-26.4)**	362	98	**27.1 (22.5-31.7)**	357	77	**21.6 (17.3-25.9)**	361	69	**19.1 (15.0-23.2)**

	0-5	115	3	**2.6 (0-5.6)**	119	5	**4.2 (0.5-7.9)**	119	1	**0.8 (0.0-2.5)**	117	6	**5.1 (1.1-9.2)**	119	4	**3.4 (0.1-6.6)**
**Northern Tanna Highlands**	5-20	216	13	**6.0 (2.8-9.2)**	218	14	**6.4 (3.1-9.7)**	221	12	**5.4 (2.4-8.4)**	216	14	**6.5 (3.2-9.8)**	220	15	**6.8 (3.5-10.2)**
	20-80	173	21	**11.6 (7.2-17.1)**	174	35	**20.1 (14.1-26.1)**	176	66	**37.9 (30.6-45.2)**	174	12	**6.9 (3.1-10.7)**	173	22	**12.7 (7.7-17.7)**

	Total	504	37	**7.3 (5.1-9.6)**	511	54	**10.6 (7.9-13.2)**	514	79	**15.4 (12.2-18.5)**	507	32	**6.3 (4.2-8.4)**	512	41	**8.0 (5.6-10.4)**

	0-5	52	1	**1.9 (0-5.8)**	52	2	**3.8 (0-9.3)**	51	0	**0.0**	51	5	**9.8 (1.4-18.3)**	52	8	**15.4 (5.2-25.5)**
**Southern Tanna**	5-20	195	3	**1.5 (0-3.3)**	188	7	**3.6 (1.0-6.2)**	192	3	**1.6 (0.0-3.3)**	195	22	**11.3 (6.8-15.8)**	194	31	**16.0 (10.8-21.2)**
	20-80	123	12	**9.8 (4.4-15.1)**	122	11	**9.0 (3.9-14.2)**	121	26	**21.5 (14.1-28.9)**	123	19	**15.5 (9.0-21.9)**	122	37	**30.3 (22.1-38.6)**

	Total	370	16	**4.3 (2.2-6.4)**	349	20	**5.4 (3.1-7.7)**	364	29	**8.0 (5.2-10.8)**	369	46	**12.5 (9.1-15.9)**	368	76	**20.7 (16.5-24.8)**

	0-5	90	1	**1.1 (0-3.3)**	90	4	**4.4 (0.1-8.8)**	90	4	**4.4 (0.1-8.7)**	90	3	**3.3 (0-7.1)**	88	3	**3.4 (0-7.3)**
**Aneityum**	5-20	218	9	**4.1 (1.5-6.8)**	218	10	**4.6 (1.8-7.4)**	219	5	**2.3 (0.3-4.3)**	219	9	**4.1 (1.5-6.8)**	210	12	**5.7 (2.6-8.9)**
	20-80	208	24	**11.5 (7.2-15.9)**	208	16	**7.7 (4.0-11.1)**	208	52	**25 (19.1-30.9)**	208	20	**9.6 (5.6-13.7)**	207	36	**17.4 (12.2-22.6)**

	Total	516	34	**6.6 (4.4-8.7)**	516	30	**5.8 (3.8-7.8)**	517	61	**11.8 (9.0-14.6)**	517	32	**6.2 (4.1-8.3)**	505	51	**10.1 (7.5-12.7)**

Number of positive individuals and seroprevalence (95% CIs) to PfMSP-1_19_, PfAMA, PfSE, PvMSP-1_19 _and PvAMA for three age groups in three settlements on Tanna and two settlements combined on Aneityum

The seroprevalence data suggest that residents in Northern Tanna have experienced the highest exposure to *P. falciparum *malaria infection, with 22% (79/358) of individuals being seropositive to one or both of the specific *P. falciparum *antigens tested. Southern Tanna had the lowest specific *P. falciparum *seroprevalence (5%, 20/369). *P. vivax *seroprevalence was also highest in Northern Tanna (30%, 107/361), followed by Southern Tanna (25%, 92/369), with the lowest seroprevalence in the Northern Tanna Highlands (11%, 54/512).

Figure 3 demonstrates the increasing seroprevalence from PfMSP-1_19 _to PfAMA with the highest seroprevalence to PfSE in each settlement. Interestingly, children under five consistently had lower seroprevalence to PfSE than to the specific antigens. This contrasts with adults who had the highest seroprevalence to PfSE in all settlements.

**Figure 4:**
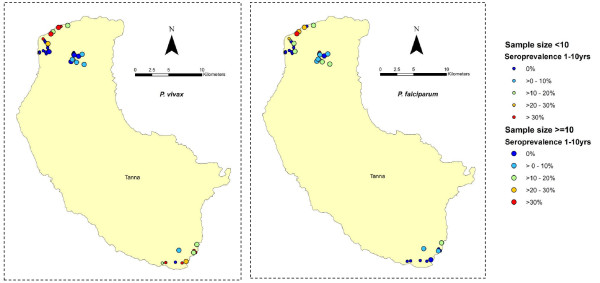
**The distribution of seroprevalence in children aged 1-10 (inclusive) in settlements on Tanna**. Seroprevalence to specific *P. falciparum *antigens (MSP-1_19 _and/or AMA-1) in children aged 1-10 (inclusive) is plotted in figure 4a and the equivalent for *P.vivax *in Figure 4b. Young children were chosen to represent local transmission as they are less likely to have travelled to areas of higher transmission. In some cases, under 10 children were sampled from a single village- these are represented by smaller circular symbols, whilst the larger symbols represent villages where more than ten, and up to 48 children were sampled.

Seroprevalence to specific antigens in children between the ages of 1 and 10 years (inclusive) by village is shown in Figure 4. It suggests that exposure is not uniform across the settlements with focal areas becoming evident in North Tanna and Southern Tanna. Unfortunately numbers were too small to allow for micro-spatial analysis.

### Changes in transmission intensity over time

**Figure 5:**
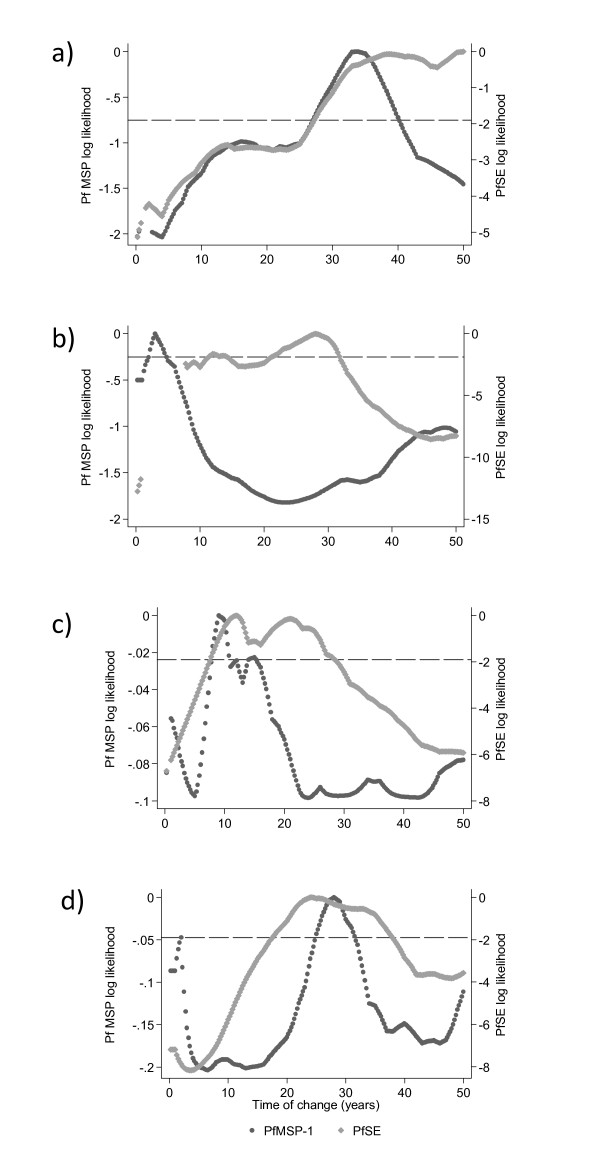
**Profile likelihood plots (PLPs) for PfMSP-1_19 _and PfSE for a) Northern Tanna b) Northern Tanna Highlands c) Southern Tanna and d) Aneityum**. Profile likelihood plots (PLPs) show the log likelihood of a catalytic conversion model allowing for a change in transmission occurring at iterative years. The maximum log likelihood is the time point at which a change in transmission is most likely to have occurred. Plots for PfAMA and the two *P. vivax *antigens showed no discernible peaks. The plots for Northern Tanna and Aneityum suggest a change in transmission occurred approximately 30 years ago, whilst the PLP for Southern Tanna suggests a more recent change between 10 and 20 years ago.

**Figure 6:**
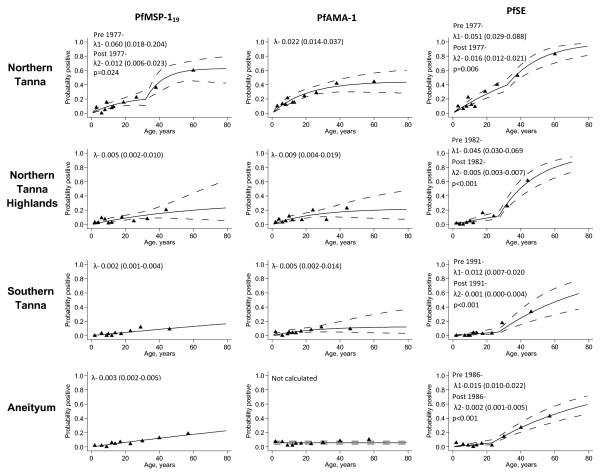
**Seroprevalence curves for all *P. falciparum *antigens for each settlement**. Seroprevalence curves represent the rate at which a community becomes seropositive to specific antigens, resulting in a seroconversion rate (SCR) or lambda (λ). A model allowing for a change in transmission intensity at a point in time has been fitted if profile likelihood plots showed a clear peak in log likelihood and if likelihood ratio tests resulted in a p < 0.05. In each graph points represent age seroprevalence (divided into deciles), unbroken lines represent maximum likelihood curves and broken lines 95% confidence intervals. Resulting lambdas (λ) and 95% confidence intervals are shown on seroprevalence curves. The Aneiytum PfAMA seroprevalence is uniformly low across all ages resulting in no seroconversion rate estimate.

Changes in transmission intensity were identified most readily for PfSE, with weak evidence in responses to the more specific PfMSP-1_19 _seroprevalence (Figure 5). No clear peaks were identified for PfAMA or for the two *P. vivax *antigens. For PfMSP-1_19_, the PLPs did not show strong evidence for a change in transmission, as demonstrated by the log likelihoods all being above the 95% confidence intervals (Figure 5) however, the MSP-1_19 _PLPs generally peaked at the same point as the PfSE plots. The maximum log likelihoods indicate that a reduction in *P. falciparum *transmission intensity occurred approximately 30 years ago in Northern Tanna (PfSE PLP: 33 years (95% CI: 27-50) and the North Tanna Highlands (PfSE PLP: 28 years (22-32 years)), 23 years ago in Aneityum (PfSE PLP: 23 years (21-32 years)) and approximately 18 years ago in Southern Tanna (PfSE PLP: 18 years (9-28 years)). The resulting SCRs suggest a 3, 9 and 12 fold decrease in transmission for North Tanna, North Tanna Highlands and Southern Tanna respectively (Figure 6). Transmission on Aneityum appears to have decreased over seven-fold. Although the decrease in transmission is not reflected in the age seroprevalence data for the specific antigens, the SCRs are very low for all settlements.

Peaks were less clear in PLP for seropositivity to *P. vivax *antigens, suggesting that *P. vivax *transmission may not have undergone a distinct reduction. A PvSE antigen was not available at the time of processing; it is possible that evidence for a change in transmission would have been detected with this antigen, as was seen with the *P. falciparum *equivalent.

**Figure 7:**
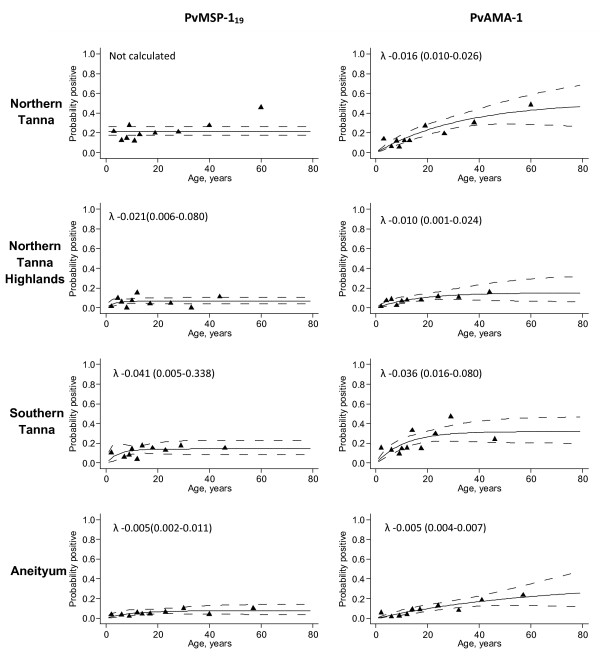
**Seroprevalence curves for all *P. vivax *antigens for each settlement**. In each graph points represent age seroprevalence by decile. Unbroken lines represent maximum likelihood curves and broken lines 95% confidence intervals. Where possible, resulting lambdas (λ) and 95% confidence intervals are shown on seroprevalence curves.

On Aneityum, it appeared that changes in age seroprevalence were more readily detected in population PfSE antibodies. The PfSE log likelihood indicated a change occurred approximately 23 years ago (18-38 years); the PfMSP-1_19 _PLP indicated a change occurred between 25 and 31 years ago, although this was not significant (Figure 7). The PvAMA seroprevalence curve for Aneityum (Figure 7) showed some evidence for a change in force of infection, although this was not reflected in the fitted model.

### Evidence for elimination?

The current SCRs for all antigens for both species were very low in Aneityum. Only the PfSE seroprevalence curve showed evidence of a clear change-point (Figure 6). However, in areas where malaria is thought to be eliminated, it is pertinent to investigate individual positive responses. Seroprevalence to specific malarial antigens in Aneityum was 10% (54/516) and 13% (65/505) for *P. falciparum *and *P. vivax *specific antigens respectively. Seropositive status was mainly restricted to older individuals. However, six children under 7 were seropositive for one or both *P. falciparum *antigens and five children under 7 were seropositive to one or both *P. vivax *antigens. Two of the children positive for *P. falciparum *antigens were reported to be one years old and antibodies may have been of maternal origin. Two children, aged 2 and aged 6, were positive to all antigens tested for both species.

The Northern settlement of Port Patrick on Aneityum had a higher proportion of seropositive individuals. Twenty-nine percent (20/70) of the individuals tested from Port Patrick were seropositive to *P. falciparum *specific antigens and 25% (17/68) were seropositive to *P. vivax *antigens, compared to 8% (34/446) and 11% (48/437) in Anel. When controlled for age group, people in Port Patrick were 5 times more likely to be *P. falciparum *seropositive and twice as likely to be *P. vivax *seropositive. Thirty-three percent (4/12) of the under 7 s (born since the 2002 epidemic) in Port Patrick were positive for *P. falciparum *antigens compared to 2% (2/91) of under 7 s in Anel (p < 0.001), while 20% (2/9) were positive for *P. vivax *antigens compared to 3% (3/90) in Anel (p < 0.001). There was no evidence of clinical infection on the island during the survey.

## Discussion

The Vanuatu government aims to eliminate malaria from Tafea province (which includes Tanna & Aneityum) in the next six years. It is thought that malaria has already been eliminated from Aneityum [3] and that transmission is confined to specific locations across Tanna. In this study, the applicability of using serological methods to provide current estimates of transmission intensity was assessed and the utility of serology to provide evidence for the cessation of transmission in Aneityum was also investigated.

Seroepidemiological studies are especially useful in low transmission settings where the sensitivity of parasite prevalence surveys is limited by the scarcity of parasite positive individuals. This study was undertaken in the dry season, and as such only three RDT positives (0.2% of the population surveyed) were found in the population. In the previous wet season, surveys using PCR methods in the same areas on Tanna found 1% prevalence of *P. falciparum *and 2.2% prevalence of *P. vivax *infection in children between 2-11 years old [22]. Both parasite-based prevalence measures, which offer a snapshot of malaria prevalence and are influenced by temporal changes, are, not surprisingly, significantly lower than the antibody prevalence data (which offers a period prevalence measure) for Tanna in this study (24%, 299/1242 *P. falciparum *and 20%, 253/1242 *P. vivax*). This extra sensitivity allows for a more detailed examination of malaria transmission.

Modelling changes in transmission intensity can give an insight into the success of interventions in a particular area. Perhaps surprisingly, changes in transmission were most readily detected using PfSE. Schizont extract contains multiple proteins some of which will be antigenic and similar between parasite species, it is, therefore, not surprising that PfSE has the highest overall seroprevalence. However, whilst adults demonstrate high seroprevalence to PfSE, seroprevalence in children is lower than for the more specific antigens, MSP-1_19 _and AMA-1. It is not clear why this should be; it may be the result of lower exposure in children resulting in lower levels of antibodies to the antigens in PfSE. This has been demonstrated in Kenya, where individuals were more likely to have antibodies to specific antigens rather than PfSE [30]. An alternative explanation is that the relative concentration of the specific antigens is lower in PfSE- this needs further investigation.

Statistical analysis of the seroprevalence profiles suggests that a significant drop in transmission occurred approximately 30 years ago. These seroprevalence curves are consistent with those described by Kaneko *et al *(submitted) using a *P. falciparum *lysate. However, analysis of country-wide case data suggests that a drop in malaria cases occurred in 1992 (17 years before this study), which was attributed to ITN distribution across the islands [24]. There are two (not mutually exclusive) potential explanations for this. Firstly, the drop in seroprevalence may indicate that the IRS campaign in place in the early 1980s successfully reduced exposure. At the time, this campaign was considered unsuccessful due to little reduction exhibited in national parasite rates. However, it is possible that it was effective in certain areas such as Northern Tanna and on Aneityum. An alternative explanation is that the reduction in seroprevalence is related to the later malaria control programme that was implemented nationwide in 1988 and in Aneityum in 1991, and which resulted in the elimination of malaria from Aneityum [3]. The observation that the step in serological data occurs approximately 10 years before this point in this scenario is possibly due to differential rates of loss of antibodies with age. Younger children who had low antibody levels when the 1988 interventions were implemented, may have lost these antibodies more rapidly than older individuals with a more established antibody response [31,32]. Clearly more information on the rates of antibody loss is required to allow a full interpretation of these data, and further work is ongoing.

The observation that immune responses to the specific antigens do not reflect the changes in transmission is perplexing. Transmission is known to be very low across Tanna and it is possible that in these conditions models for assessing for an increase in seropositivity with age may be inappropriate. Several of the age stratified curves demonstrate little to no increase in seroprevalence with age. In areas where transmission has been consistently low for many years a loss in seropositivity in older individuals may result in uniform seropositivity across the population; masking any change-point in transmission. This is clearly not the case for the multi-antigenic PfSE- one might expect an increased chance that antibodies to this conglomeration of antigens have longer half lives, although this was not observed in a longitudinal study in Thailand [33]. In a situation where age seroprevalence reveals little information, it may be more pertinent to investigate antibody titres and to focus on children born since interventions have been in place. This work is ongoing.

It is important to understand the dynamics in the transmission of different species in areas where more than one species of *Plasmodium *are transmitted. Serological measures have allowed us to successfully distinguish between areas where *P. falciparum *and *P. vivax *malaria are transmitted. Current SCRs are low for both species but consistently higher for *P. vivax *in all areas. Seropositivity to *P. vivax *antigens was much higher in younger individuals, suggesting either that transmission of this species has been higher than *P. falciparum *transmission over the past 10 years, or that preferential infection of younger age groups, as has been reported previously [34], results in a different age seroprevalence curve than is found with *P. falciparum *seroprevalence. Additionally, morbidity data from previous studies in Vanuatu [35] and Papua New Guinea [36] show that clinical immunity to *P. vivax *builds up faster than for *P. falciparum *which could explain the seemingly high seroprevalence in younger children. The high proportion of *P. vivax *seropositive individuals in younger age groups in Northern Tanna made the data difficult to fit the conventional seroconversion model. A similar age seroprevalence profile would be expected in areas where there has been an epidemic as the result of a defined period of exposure in all ages of the population. However, the effect of possible presence of hypnozoites in *P. vivax *infections has not previously been studied and we do not know how infections that are the result of hypnozoites (i.e. relapses) influence serological profiles. Previous data has shown that *P. falciparum *is more successfully reduced by interventions than *P. vivax*, due to the limited effects interventions have on relapses, and due to differences in biting behaviour by mosquitoes infected with each species [37]. The profile likelihood plots do not indicate a clear change in transmission intensity based on seroprevalence of *P. vivax *antibodies. The drop in *P. vivax *transmission may not have been as pronounced or sudden as the *P. falciparum *reduction, in which case, a step in the seroprevalence curves would not be evident. *P. vivax *prevalence tends to be higher in areas of low transmission intensity [38], and an increase in *P. vivax *cases has been reported in areas where *P. falciparum *transmission appears to be on the decline [39,40]. However, on Aneityum clear evidence of a change in transmission for *P. vivax *was seen in seroprevalence data by Kaneko *et al *(submitted); this data also indicated a change in transmission had occurred in the early 1980s. This study used a lysed *P.vivax *preparation as antigen which, as for the *P. falciparum *equivalent, may be more likely to detect changes in a polymorphic antibody response.

The *P. vivax *epidemic in 2002 may also have had an adverse effect on the seroprevalence curves to *P. vivax *antigens in Aneityum. There have been no reported cases since 2002, and whilst seropositivity on this island is mainly restricted to adults, low seropositivity in children under 7 suggests that there may still be residual transmission in the area. However, importantly, four of the 11 children seropositive to any antigen (falciparum or vivax) were classified as aged one. Birthdays and ages are not generally recorded in Aneityum, and it is possible these children were younger than one and, therefore, the antibodies detected could represent residual maternal antibodies. Alternatively, children may have been exposed to malaria whilst away from the island. Limited information was collected on travel history during the survey so this possible explanation cannot be confirmed. Monitoring of cases on Aneityum is very thorough, with everyone returning to the island tested for infection via thick and thin slide films. However, slide reading has limited sensitivity, and it is possible that some people may have arrived carrying infection. PCR methods detect parasites more sensitively and it may be pertinent to check for sub-patent infections amongst the inhabitants of Aneityum. Imported malaria can remain a problem as long as Anopheline mosquitoes are present on the island. This highlights the importance of continued monitoring and surveillance on islands where elimination has been achieved [41], and the recording of accurate travel history information.

An alternative explanation for the observed seropositivity in young children is the presence of antibodies cross-reacting with antigens from other infectious agents [42-44]. Detecting antibodies to specific recombinant antigens using ELISA, rather than the less specific immunofluoresence antibody test (IFAT) minimizes this danger. However, a few studies have suggested the cross-reactive potential of PfAMA with Toxoplasma antigens [45,46], although this infection is reportedly not prevalent in Vanuatu.

## Conclusion

Malaria transmission is notably heterogeneous, and this will become more pronounced as transmission decreases. For malaria elimination, areas where residual transmission is occurring need to be identified in order to target interventions to communities where they are most needed. In Vanuatu, interventions have been very successful at reducing MTI over the past 20 years. However, areas of higher transmission were identified on Tanna during the 2008 survey [22]. Serological analysis and monitoring of these areas can offer further insight into the dynamics of malaria transmission. The additional sensitivity afforded by the longevity of antibody responses also allows an examination of the success of control measures also in confirming the cessation of transmission. Serological measures are robust and high throughput and importantly, remain sensitive in very low transmission settings.

## Competing interests

The authors declare that they have no competing interests.

## Authors' contributions

JC carried out the survey, the serological experiments, and the serological analysis and wrote the first draft of the manuscript. HR developed the study protocol, led the field survey and produced the maps for the serological analysis with support from AC. JI participated in field operations, data and laboratory specimen collection and conducted serological assays. MK supported field team operations, coordinated laboratory procedures and serological testing. GT conceived the study, participated in its design and supervised field coordination. JM, AV, AC and CD conceived the study, participated in its design and implementation and supervised fieldwork, laboratory investigations, data analysis and manuscript development. All authors read and approved the final manuscript.

## References

[B1] Feachem RGA Phillips AA Targett GA Shrinking the Malaria Map: A prospectus on Malaria Elimination 2009 The Global Health Group

[B2] Bousema T Drakeley C Gesase S Hashim R Magesa S Mosha F Otieno S Carneiro I Cox J Msuya E Kleinschmidt I Maxwell C Greenwood B Riley E Sauerwein R Chandramohan D Gosling R Identification of hot spots of malaria transmission for targeted malaria control J Infect Dis 2010 201 1764 1774 10.1086/652456 20415536

[B3] Kaneko A Taleo G Kalkoa M Yamar S Kobayakawa T Bjorkman A Malaria eradication on islands Lancet 2000 356 1560 1564 10.1016/S0140-6736(00)03127-5 11075770

[B4] Teklehaimanot HD Teklehaimanot A Kiszewski A Rampao HS Sachs JD Malaria in Sao Tome and principe: on the brink of elimination after three years of effective antimalarial measures Am J Trop Med Hyg 2009 80 133 140 19141851

[B5] Bruce-Chwatt LJ Draper CC Konfortion P Seroepidemiological evidence of eradication of malaria from Mauritius Lancet 1973 2 547 551 10.1016/S0140-6736(73)92361-1 4125305

[B6] Smith DL Hay SI Endemicity response timelines for *Plasmodium falciparum *elimination Malar J 2009 8 87 10.1186/1475-2875-8-87 19405974 PMC2686731

[B7] Kelly-Hope LA McKenzie FE The multiplicity of malaria transmission: a review of entomological inoculation rate measurements and methods across sub-Saharan Africa Malar J 2009 8 19 10.1186/1475-2875-8-19 19166589 PMC2656515

[B8] Robert V Le Goff G Andrianaivolambo L Randimby FM Domarle O Randrianarivelojosia M Raharimanga V Raveloson A Ravaonjanahary C Ariey F Moderate transmission but high prevalence of malaria in Madagascar Int J Parasitol 2006 36 1273 1281 10.1016/j.ijpara.2006.06.005 16842796

[B9] Elissa N Migot-Nabias F Luty A Renaut A Toure F Vaillant M Lawoko M Yangari P Mayombo J Lekoulou F Tshipamba P Moukagni R Millet P Deloron P Relationship between entomological inoculation rate, *Plasmodium falciparum *prevalence rate, and incidence of malaria attack in rural Gabon Acta Trop 2003 85 355 361 10.1016/S0001-706X(02)00266-8 12659973

[B10] Corran P Coleman P Riley E Drakeley C Serology: a robust indicator of malaria transmission intensity? Trends Parasitol 2007 23 575 582 10.1016/j.pt.2007.08.023 17988945

[B11] Voller A Bruce-Chwatt LJ Serological malaria surveys in Nigeria Bull World Health Organ 1968 39 883 897 4893493 PMC2554577

[B12] Cornille-Brogger R Mathews HM Storey J Ashkar TS Brogger S Molineaux L Changing patterns in the humoral immune response to malaria before, during, and after the application of control measures: a longitudinal study in the West African savanna Bull World Health Organ 1978 56 579 600 365386 PMC2395642

[B13] Voller A Cornille-Brogger R Storey J Molineaux L A longitudinal study of Plasmodium falciparum malaria in the West African savannah using the ELISA technique Bull World Health Organ 1980 58 429 438 6998590 PMC2395907

[B14] Draper CC Lelijveld JL Matola YG White GB Malaria in the Pare area of Tanzania. IV. Malaria in the human population 11 years after the suspension of residual insecticide spraying, with special reference to the serological findings Trans R Soc Trop Med Hyg 1972 66 905 912 10.1016/0035-9203(72)90126-5 4569251

[B15] Ambroise-Thomas P Wernsdorfer WH Grab B Cullen J Bertagna P Etude sero-epidemiologique longitudinale sur la paludisme en Tunisie Bull Organ Mond Sante 1976 54 355 367 PMC2366475 1088349

[B16] Bruce-Chwatt LJ Draper CC Avramidis D Kazandzoglou O Sero-epidemiological surveillance of disappearing malaria in Greece J Trop Med Hyg 1975 78 194 200 772232

[B17] Drakeley C Cook J Chapter 5. Potential contribution of sero-epidemiological analysis for monitoring malaria control and elimination: historical and current perspectives Adv Parasitol 2009 69 299 352 full_text 19622411 10.1016/S0065-308X(09)69005-9

[B18] Drakeley C Corran P Coleman PG Tongren JE McDonald S Carneiro I Malima R Lusingu JPA Manjurano A Nkya WM Lemnge MM Cox J Reyburn H Riley EM Estimating medium and long term trends in malaria transmission using serological markers of malaria exposure Proc Natl Acad Sci USA 2005 102 5108 5113 10.1073/pnas.0408725102 15792998 PMC555970

[B19] Corran PH Cook J Lynch C Leendertse H Manjurano A Griffin J Cox J Abeku T Bousema T Ghani AC Drakeley C Riley EM Dried blood spots as a source of anti-malarial antibodies for epidemiological studies Malar J 2008 7 195 10.1186/1475-2875-7-195 18826573 PMC2567984

[B20] Stewart L Gosling R Griffin J Gesase S Campo J Hashim R Masika P Mosha J Bousema T Shekalaghe S Cook J Corran P Ghani A Riley EM Drakeley C Rapid assessment of malaria transmission using age-specific sero-conversion rates PLoS One 2009 4 e6083 10.1371/journal.pone.0006083 19562032 PMC2698122

[B21] Williams GS Mweya C Stewart L Mtove G Reyburn H Cook J Corran PH Riley EM Drakeley CJ Immunophoretic rapid diagnostic tests as a source of immunoglobulins for estimating malaria sero-prevalence and transmission intensity Malar J 2009 8 168 10.1186/1475-2875-8-168 19624812 PMC2720984

[B22] Reid H Vallely A Taleo G Tatem AJ Kelly G Riley I Harris I Iata H Yama S Clements A Baseline spatial distribution of malaria prior to an elimination program in Vanuatu Malar J 2010 in press 10.1186/1475-2875-9-150 PMC2893196 20525209

[B23] Atkinson JA Fitzgerald L Toaliu H Taleo G Tynan A Whittaker M Riley I Vallely A Community participation for malaria elimination in Tafea Province, Vanuatu: Part I. Maintaining motivation for prevention practices in the context of disappearing disease Malar J 2010 9 93 10.1186/1475-2875-9-93 20380748 PMC2873527

[B24] Chaves LF Kaneko A Taleo G Pascual M Wilson ML Malaria transmission pattern resilience to climatic variability is mediated by insecticide-treated nets Malar J 2008 7 100 10.1186/1475-2875-7-100 18518983 PMC2443810

[B25] Gray DJ Forsyth SJ Li RS McManus DP Li Y Chen H Zheng F Williams GM An innovative database for epidemiological field studies of neglected tropical diseases PLoS Negl Trop Dis 2009 3 e413 10.1371/journal.pntd.0000413 19478833 PMC2680951

[B26] Nagelkerke NJ Borgdorff MW Kim SJ Logistic discrimination of mixtures of M. tuberculosis and non-specific tuberculin reactions Stat Med 2001 20 1113 1124 10.1002/sim.745 11276040

[B27] Hardelid P Williams D Dezateux C Tookey PA Peckham CS Cubitt WD Cortina-Borja M Analysis of rubella antibody distribution from newborn dried blood spots using finite mixture models Epidemiol Infect 2008 1 9 10.1017/S0950268808000393 PMC2870791 18294427

[B28] Rota MC Massari M Gabutti G Guido M De Donno A Atti ML Measles serological survey in the Italian population: Interpretation of results using mixture model Vaccine 2008 26 4403 4409 10.1016/j.vaccine.2008.05.094 18585420

[B29] SPC Secretariat of the Pacific Community: Oceania Population Data Sheet 2000

[B30] Osier FH Fegan G Polley SD Murungi L Verra F Tetteh KK Lowe B Mwangi T Bull PC Thomas AW Cavanagh DR McBride JS Lanar DE Mackinnon MJ Conway DJ Marsh K Breadth and magnitude of antibody responses to multiple *Plasmodium falciparum *merozoite antigens are associated with protection from clinical malaria Infect Immun 2008 76 2240 2248 10.1128/IAI.01585-07 18316390 PMC2346713

[B31] Akpogheneta OJ Duah NO Tetteh KK Dunyo S Lanar DE Pinder M Conway DJ Duration of naturally acquired antibody responses to blood-stage *Plasmodium falciparum *is age dependent and antigen specific Infect Immun 2008 76 1748 1755 10.1128/IAI.01333-07 18212081 PMC2292892

[B32] Kinyanjui SM Conway DJ Lanar DE Marsh K IgG antibody responses to *Plasmodium falciparum *merozoite antigens in Kenyan children have a short half-life Malar J 2007 6 82 10.1186/1475-2875-6-82 17598897 PMC1920526

[B33] Wipasa J Suphavilai C Okell LC Cook J Corran PH Thaikla K Liewsaree W Riley EM Hafalla JC Long-lived antibody and B cell memory responses to the human malaria parasites, *Plasmodium falciparum *and *Plasmodium vivax* PLoS Pathog 2010 6 e1000770 10.1371/journal.ppat.1000770 20174609 PMC2824751

[B34] Price RN Tjitra E Guerra CA Yeung S White NJ Anstey NM Vivax malaria: neglected and not benign Am J Trop Med Hyg 2007 77 79 87 18165478 PMC2653940

[B35] Maitland K Williams TN Bennett S Newbold CI Peto TE Viji J Timothy R Clegg JB Weatherall DJ Bowden DK The interaction between *Plasmodium falciparum *and *P. vivax *in children on Espiritu Santo island, Vanuatu Trans R Soc Trop Med Hyg 1996 90 614 620 10.1016/S0035-9203(96)90406-X 9015495

[B36] Lin E Kiniboro B Gray L Dobbie S Robinson L Laumaea A Schopflin S Stanisic D Betuela I Blood-Zikursh M Siba P Felger I Schofield L Zimmerman P Mueller I Differential patterns of infection and disease with *P. falciparum *and *P. vivax *in Young Papua New Guinean children PLoS One 2010 5 e9047 10.1371/journal.pone.0009047 20140220 PMC2816213

[B37] Bockarie MJ Dagoro H Are insecticide-treated bednets more protective against *Plasmodium falciparum *than *Plasmodium vivax*-infected mosquitoes? Malar J 2006 5 15 10.1186/1475-2875-5-15 16504027 PMC1388224

[B38] Mendis K Sina BJ Marchesini P Carter R The neglected burden of *Plasmodium vivax *malaria Am J Trop Med Hyg 2001 64 97 106 11425182 10.4269/ajtmh.2001.64.97

[B39] Childs DZ Cattadori IM Suwonkerd W Prajakwong S Boots M Spatiotemporal patterns of malaria incidence in northern Thailand Trans R Soc Trop Med Hyg 2006 100 623 631 10.1016/j.trstmh.2005.09.011 16406037

[B40] da Silva-Nunes M Codeco CT Malafronte RS da Silva NS Juncansen C Muniz PT Ferreira MU Malaria on the Amazonian frontier: transmission dynamics, risk factors, spatial distribution, and prospects for control Am J Trop Med Hyg 2008 79 624 635 18840755

[B41] D'Ortenzio E Sissoko D Dehecq JS Renault P Filleul L Malaria imported into Reunion Island: is there a risk of re-emergence of the disease? Trans R Soc Trop Med Hyg 2009 19914673 10.1016/j.trstmh.2009.10.008

[B42] Naus CW Jones FM Satti MZ Joseph S Riley EM Kimani G Mwatha JK Kariuki CH Ouma JH Kabatereine NB Vennervald BJ Dunne DW Serological responses among individuals in areas where both schistosomiasis and malaria are endemic: cross-reactivity between *Schistosoma mansoni *and *Plasmodium falciparum* J Infect Dis 2003 187 1272 1282 10.1086/368361 12696007

[B43] Mutapi F Roussilhon C Mduluza T Druilhe P Anti-malaria humoral responses in children exposed to *Plasmodium falciparum *and *Schistosoma haematobium* Mem Inst Oswaldo Cruz 2007 102 405 409 10.1590/S0074-02762007005000046 17568947

[B44] Abramo C Fontes CJ Krettli AU Cross-reactivity between antibodies in the sera of individuals with leishmaniasis, toxoplasmosis, and Chagas' disease and antigens of the blood-stage forms of *Plasmodium falciparum *determined by indirect immunofluorescence Am J Trop Med Hyg 1995 53 202 205 7677225 10.4269/ajtmh.1995.53.202

[B45] Donahue CG Carruthers VB Gilk SD Ward GE The Toxoplasma homolog of Plasmodium apical membrane antigen-1 (AMA-1) is a microneme protein secreted in response to elevated intracellular calcium levels Mol Biochem Parasitol 2000 111 15 30 10.1016/S0166-6851(00)00289-9 11087913

[B46] Gaffar FR Yatsuda AP Franssen FF de Vries E Erythrocyte invasion by *Babesia bovis *merozoites is inhibited by polyclonal antisera directed against peptides derived from a homologue of *Plasmodium falciparum *apical membrane antigen 1 Infect Immun 2004 72 2947 2955 10.1128/IAI.72.5.2947-2955.2004 15102807 PMC387893

